# Pseudoaneurysm of the brachial artery in an infant due to vaccination: a case report

**DOI:** 10.1186/s12887-022-03793-2

**Published:** 2023-01-05

**Authors:** Hai-fei Shi, Shuai Yuan, Ke-jiong Liang, Po Ye, Hu Yang

**Affiliations:** 1 Department of Orthopedics, The First Affiliated Hospital, Zhejiang University School of Medicine, 79# Qingchun Road, Hangzhou, 310003 China; 2 Department of Echocardiography and Vascular Ultrasound Center, The First Affiliated Hospital, Zhejiang University School of Medicine, Hangzhou, China

**Keywords:** Brachial artery, Complication, Infant, Pseudoaneurysm, Vaccination, Case report

## Abstract

**Background:**

Pseudoaneurysm is a known complication of penetrating arterial injuries such as catheterization, gunshot wounds, and open fractures. Vaccination is an effective method for preventing multiple, serious, infectious diseases in children. Common adverse reactions related to vaccination include fever, swelling, redness, and pain. Brachial pseudoaneurysm after vaccination has not been previously reported.

**Case presentation:**

Herein we describe a novel case of brachial pseudoaneurysm after vaccination in a child aged 1 year and 3 months. A pulsatile mass was formed in the medial left arm of the infant 10 days after vaccination at a community hospital and gradually grew larger. Preoperative images depicted an eccentric aneurysm in the brachial artery and a swirling flow pattern in the mass. The pseudoaneurysm was excised, and vein graft interpositioning was successfully performed. There were no short-term or long-term complications during the follow-up period.

**Conclusions:**

Brachial pseudoaneurysm is a rare complication of vaccination via intramuscular injection. Medical staff should avoid puncture wounds to the brachial artery during vaccination, especially in infants.

## Background

Pseudoaneurysm is caused by a wide variety of paravascular infectious, inflammatory, and traumatic processes such as tuberculous lymph nodes, mycotic infection [1], adjacent abscess [2], pancreatitis [3], angiography, artery puncture [4], and endovascular surgeries. Pseudoaneurysms as a result of trauma reportedly occur in less than 0.5% of patients [5]. Pseudoaneurysms of the brachial artery are even rarer and are usually a result of either trauma or iatrogenic injury.

Vaccination is a necessary and safe method for preventing numerous, serious infectious diseases in infants. There are three main types of injected vaccination: intradermal, subcutaneous, and intramuscular. Adverse reactions related to vaccination include fever, redness, swelling, local pain, headache, fatigue, nausea, vomiting, diarrhea, allergic response, and systemic infection. Pseudoaneurysm arising from vaccination has never been reported. Herein, we report an unusual pseudoaneurysm of the brachial artery in an infant due to vaccination. In addition to the age of the patient, the case is notable due to the associated mechanism of injury—vaccination. This case report describes a new, possible, adverse reaction during vaccination, especially in pediatric patients.

## Case presentation

A child aged 1 year and 3 months was presented with a pulsatile mass in the proximal medial left arm. Two months prior, the infant had been vaccinated against *Haemophilus influenzae* type b (Hib) at a community hospital. The vaccine had been administered in the proximal lateral left arm via intramuscular injection, and there were no complications at the time of vaccination. Ten days later, the patient exhibited a pulsatile mass in the proximal medial left arm. Thereafter, the mass gradually became larger. With increasing prominence of the mass, the parents visited our hospital for treatment. The patient did not undergo any related treatment before admission.

**Fig. 1:**
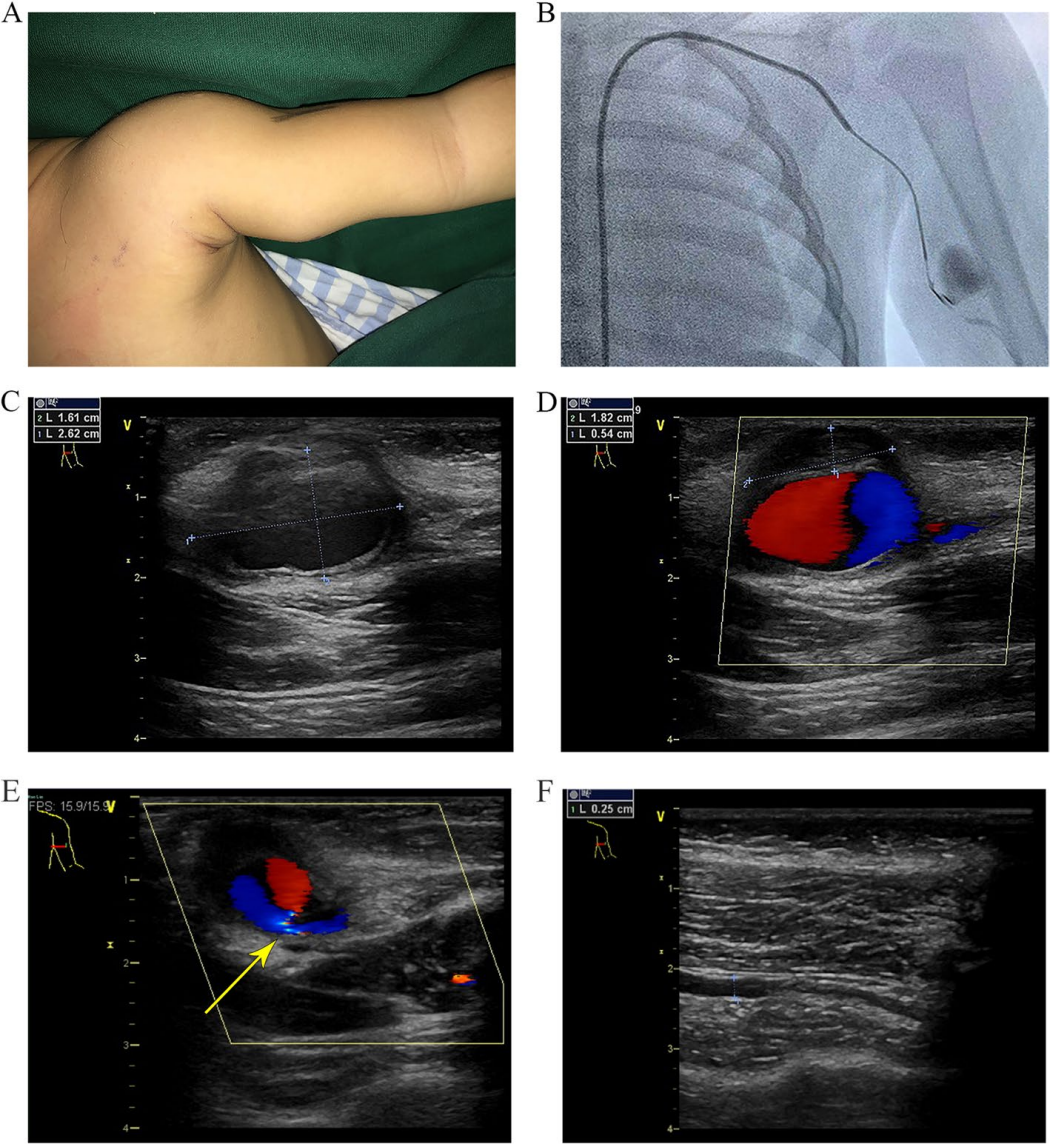
Preoperative examination of the patient. **A** A mass can be seen under the skin in the medial left arm. **B** Digital subtraction angiography revealed an eccentric aneurysm in the brachial artery. **C** Doppler ultrasonography revealed a cystic mass measuring 2.62 cm × 1.61 cm. **D** Swirling flow pattern in the mass was confirmed with thrombus adherent to the wall of the mass. **E** Preoperative ultrasound measurement of pseudoaneurysm neck is very thin, ~ 0.2 cm in length. **F** Preoperative ultrasound measurement of normal segment brachial artery diameter—0.25 cm

The infant was well developed, and the results of cardiopulmonary examination were normal. Physical examination revealed a non-tender, pulsatile mass in the medial left arm measuring 3 cm × 2 cm, with a clear boundary. The skin on the surface of the mass was intact, without redness or damage (Fig. 1A). Peripheral pulses were fully detectable, and there were no clinical signs of numbness in the fingertips. Left upper limb motion was normal. No trauma had been encountered after vaccination; no invasive procedure had been performed; and there was no family history of aneurysm or congenital cardiovascular disorders.

Electrocardiography, chest roentgenography, and echocardiography did not reveal any evidence of cardiac disease. Blood tests were normal. Digital subtraction angiography (DSA) revealed an eccentric aneurysm in the brachial artery (Fig. 1B). Doppler ultrasonography revealed a cystic mass measuring 2.62 cm × 1.61 cm (Fig. 1C). The cystic mass had arterial flow with a neck that could be traced back to the brachial artery. A swirling flow pattern was evident in the mass, and there was a thrombus adherent to the wall of the mass (Fig. 1D). The wall of the mass was thicker than the artery wall. Hence, the diagnosis was brachial pseudoaneurysm. This patient’s pseudoaneurysm communicated with the brachial artery via a short neck (Fig. 1E), and the aneurysm had a high degree of enlargement compared to the normal diameter (Fig. 1F). Although the patient was only 1 year and 3 months old, we decided to perform surgery to prevent the pseudoaneurysm from getting bigger or bursting.

**Fig. 2:**
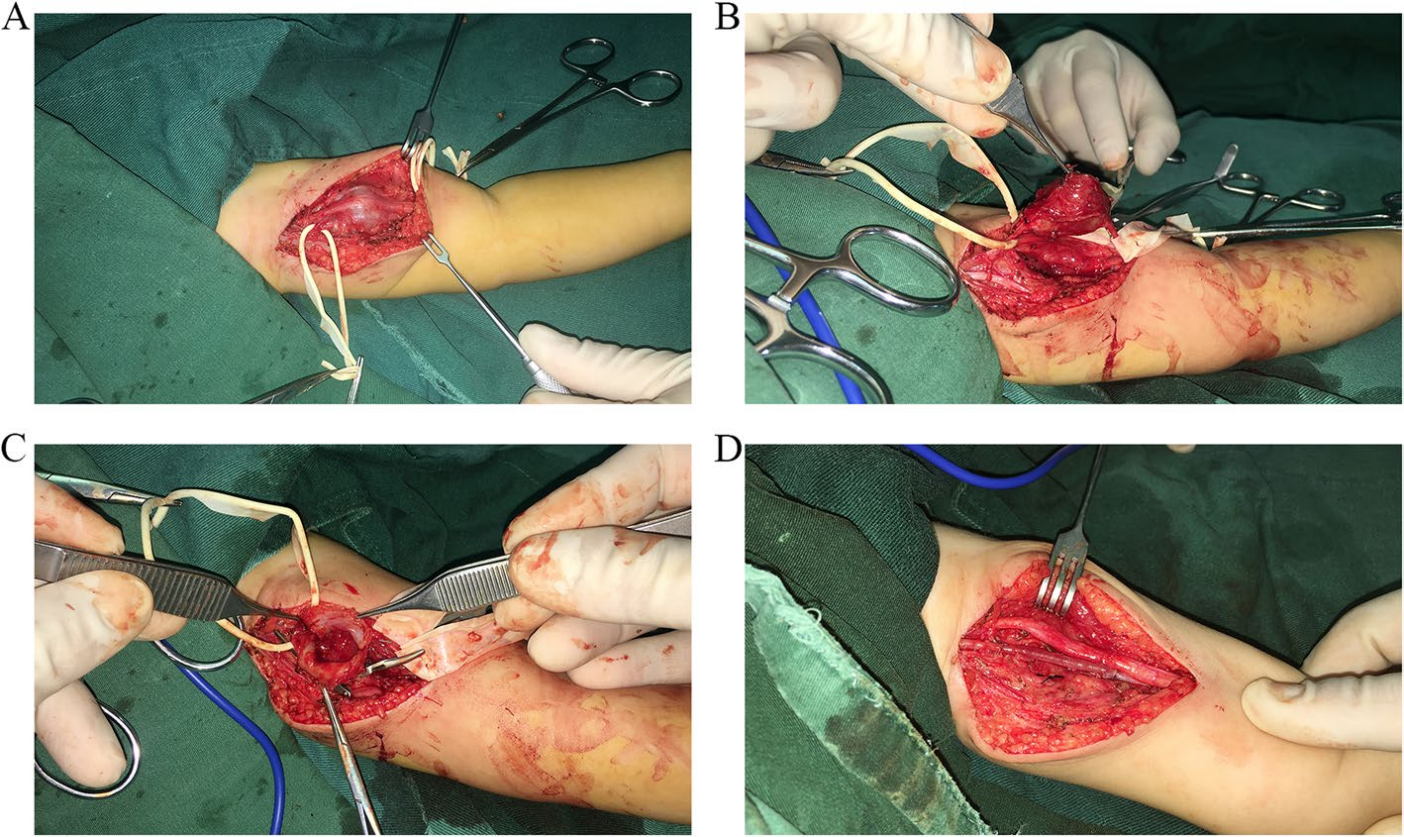
Surgical findings. **A** The mass was just under the median nerve. The median nerve was squeezed and stretched by the mass. **B** The mass was carefully separated and connected to the brachial artery. **C** The mass was dissected through the surface, with a mural thrombus found adherent to the wall. **D** A 3-cm-long reversed graft of the great saphenous vein was interposed between the cut ends of the brachial artery

Open surgery was conducted under general anesthesia. The patient was placed in the supine position with the arm abducted. An incision was made along the medial upper arm. A cystic and eccentric brachial aneurysm was detected just under the median nerve, which was squeezed and stretched by the aneurysm (Fig. 2A). The aneurysm was carefully separated and excised under control of the proximal and distal brachial artery (Fig. 2B), and the nerve compression was relieved during this process. The mass was dissected through the surface and contained blood and a mural thrombus adherent to its wall (Fig. 2C). On close inspection, a faint trace of trauma to the wall was evident in the tissue surrounding the brachial artery. A 3-cm-long reversed graft from the great saphenous vein was interposed between the cut ends of the brachial artery (Fig. 2D). The excised mass was evaluated histopathologically. According to the pathological report, the excised cystic mass had an outer wall formed by fibrous tissue hyperplasia, which was consistent with the diagnosis of pseudoaneurysm.

**Fig. 3:**
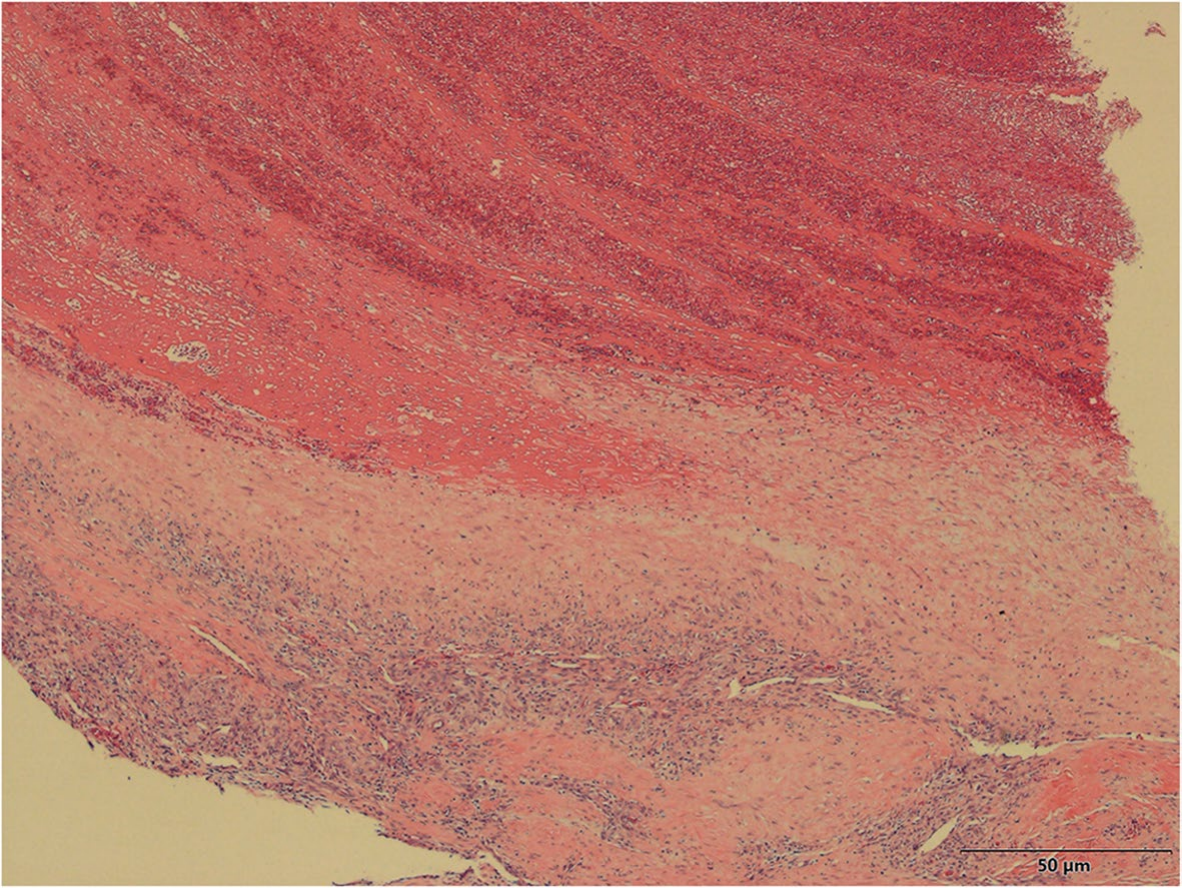
Hematoxylin-eosin staining of the mass (scale bar = 50 μm, 40X magnification of camera lens). The result shows inflammatory infiltration, hyalinization, and regions of hematoma in addition to the vessel wall lumen

**Fig. 4:**
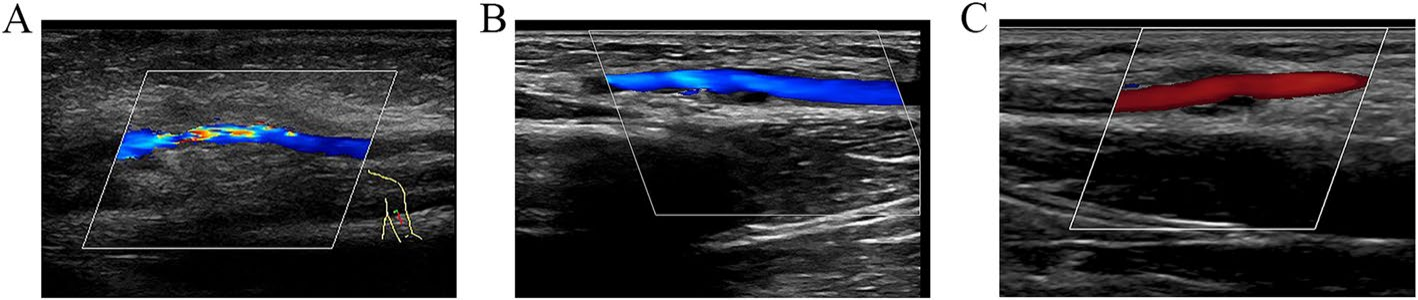
Postoperative Doppler ultrasonography results. **A** Partial stenosis and local inflammation around the reconstructed artery was seen at 1 week after surgery. **B** The artery stenosis and inflammation around the artery disappeared at 5 months. **C** Homogeneous and stable blood flow in the reconstructed brachial artery was seen without stenosis at 1 year

The surgery was successful. Dressings were changed every 2 days. The patient did not experience any complications during hospitalization. Histopathologic evaluation of the mass indicated inflammatory infiltration, hyalinization, and regions of hematoma beside the vessel wall lumen (Fig. 3), which confirmed injury to the vessel. Injury to the vascular wall led to the formation of a pseudoaneurysm. Blood flow through the reconstructed artery was smooth. Postoperative Doppler ultrasonography at 1 week revealed 100% patency of the brachial artery except for partial stenosis and local inflammation around the reconstructed area (Fig. 4A). The peak systolic velocity of the reconstructed artery was substantially faster than that of the distal ulnar artery and normal brachial artery at 1 week.

**Fig. 5:**
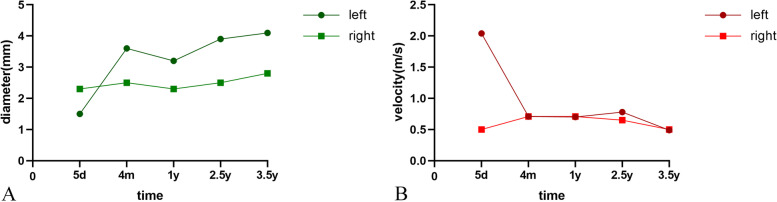
Follow-up ultrasound measurements of the graft segment vein and right healthy brachial artery at 5 days, 4 months, 1 year, 2.5 years, and 3.5 years after surgery. **A** Ultrasound measurement of graft diameter; **B** ultrasound measurements of blood velocity

The patient was presented regularly for follow-up evaluation. No numbness, muscle atrophy, or ischemia were identified. Examination of the reconstructed artery at 5 months indicated that artery stenosis and inflammation around the artery had disappeared (Fig. 4B), and peak systolic velocity had returned to normal. The parameters of the artery were essentially unchanged at 1 year of age (Fig. 4C). No sequelae were reported during the late period. The parents were satisfied with the result. Based on the latest follow-up results, we mapped the postoperative changes of the grafted vessels. The diameter of the graft segment was dilated compared to the right healthy brachial artery (Fig. 5A), while the blood flow velocity was basically the same as the right side (Fig. 5B).

## Discussion and conclusions

Hib vaccine prevents meningitis, pneumonia, epiglottitis, and other serious infections caused by the Hib bacterium. It is usually injected into the deltoid muscle. According to the guidelines for Hib vaccination from the Center for Disease Control, common adverse reactions include swelling, redness, and pain; systemic reactions such as fever and irritability are infrequent, and serious reactions are rare. In a report on adverse events after Hib vaccination, other outcomes included pyrexia, vomiting, convulsions, irritability, intussusception, diarrhea, crying, hypotonia, lethargy, and apnea [6]. Pseudoaneurysm as an adverse reaction associated with Hib vaccination has never been reported.

Aneurysm refers to the weakening of an artery wall that creates a bulge or distention of the artery. Most aneurysms are asymptomatic and are not dangerous. At their most severe stage, however, some can rupture, leading to life-threatening, internal bleeding. Aneurysms occurring in children are rare and usually occur in the thoracic or abdominal aorta [7]. Nonaortic arterial aneurysms in children are rarer and have been associated with neurofibromatosis [8], Kawasaki disease [9], periarteritis nodosa [10], and giant cell arteritis [11].

Pseudoaneurysm is a collection of flowing blood that communicates with the arterial lumen and is filled only by the adventitia or surrounding soft tissue. It is a known complication of penetrating arterial injuries such as catheterization, gunshot wounds, and fractures [12]. It may take days, months, or even years to become symptomatic or be detected clinically. Symptoms are usually limited to the mass effect of the lesion and can include tenderness, pain, and swelling. Complications related to peripheral artery pseudoaneurysm include local pain, rupture, neuropathy, local limb ischemia, and even limb loss [13]. In medical practice, iatrogenic arterial injury should be avoided as much as possible to reduce the occurrence of serious complications such as pseudoaneurysm. Although vaccination does not typically affect the arteries, the operator’s knowledge of the anatomy of the vaccination site is still important. In particular, because infants are small, the distance between the brachial artery and the deltoid muscle is relatively short. Therefore, it is necessary to strengthen the technical training of vaccination operators to avoid related iatrogenic injuries.

Pseudoaneurysm can be diagnosed via Doppler ultrasonography, computed tomography angiography, DSA, and magnetic resonance angiography [14]. Doppler ultrasonography can reveal the presence of blood flow or thrombi within the pseudoaneurysm, the size of the neck of the pseudoaneurysm, the integrity of adjacent vessels, and the presence of loculations. Moreover, it is a useful modality during patient follow-up after treatment because it is rapid, convenient, and inexpensive. Computed tomography angiography and DSA can depict the vasculature in different ways, but intravascular contrast medium is required. Magnetic resonance angiography is also a preferred method for diagnostic evaluations of pediatric vascular diseases due to its high image resolution, lack of arterial invasion, and absence of radiation exposure. However, it is comparatively expensive and inconvenient.

Pseudoaneurysm treatments include conservative measures, sonographically guided compression, embolization, and open surgery. Sonographically guided compression was introduced in 1991 and involves the placement of an ultrasound probe directly over the neck of the pseudoaneurysm in conjunction with the application of prolonged compression, thereby producing vascular stasis within the lumen and promoting thrombosis [15]. Although it is noninvasive and its success rate is approximately 90%, the procedure is often painful for patients and uncomfortable for operators, time-consuming, and occasionally unsuccessful [16]. Sonographically guided thrombin injection was used extensively after its introduction in 1997 because of its high success rate, minimal patient discomfort, and immediate results. It involved percutaneous injection of thrombin into the lumen under sonographic guidance. Although success rates ranged from 91 to 100%, the fear of embolic and thrombotic complications hindered the primary use of this method [17]. It was suggested that compression should be used as the first-line treatment and that thrombin injection should be reserved as a backup. Open surgery includes resection, ligation, reanastomosis, or vein graft interpositioning [18].

Vein graft interpositioning was preferred in the current case due to the size and rapid expansion of the pseudoaneurysm. Fortunately, the operation was performed in time, which enabled the squeezed median nerve to be released, thus avoiding further damage. Long-term follow-up indicated that the surgery was effective and without obvious complications.

In the present case, preoperative image reports, intraoperative surgical findings, and postoperative pathological examination all confirmed the existence of brachial pseudoaneurysm. Because the infant did not suffer from any hereditary disease and had not experienced any other iatrogenic or traumatic injury, we speculated that vaccination into the deltoid muscle had accidentally punctured the brachial artery, leading to the formation of a pulsative mass 10 days later. Although rare, this can happen, resulting in pseudoaneurysm. Fortunately, the surgery was timely; otherwise, sensory and motor dysfunction could have occurred while the median nerve was severely squeezed. The infant recovered well with no complications.

Herein, we have described a case of brachial artery pseudoaneurysm formation after vaccination in an infant, in whom resection of the pseudoaneurysm and vein graft interpositioning were successful. We emphasize that although vaccination can rarely lead to pseudoaneurysm, it is mostly safe. Efforts must be made to avoid such complications during vaccination.

## Data Availability

The materials used during the current study are available from the corresponding author on reasonable request.

## Abbreviations

Hib: *Haemophilus influenzae* Type b
DSA: Digital subtraction angiography
US: Ultrasonography
PSV: Peak systolic velocity
CTA: Computed tomography angiography
MRA: Magnetic resonance angiography
